# A GMC Oxidoreductase GmcA Is Required for Symbiotic Nitrogen Fixation in *Rhizobium leguminosarum* bv. *viciae*

**DOI:** 10.3389/fmicb.2020.00394

**Published:** 2020-03-24

**Authors:** Qian Zou, Sha Luo, Hetao Wu, Donglan He, Xiaohua Li, Guojun Cheng

**Affiliations:** Hubei Provincial Engineering and Technology Research Center for Resources and Utilization of Microbiology, College of Life Sciences, South-Central University for Nationalities, Wuhan, China

**Keywords:** *Rhizobium leguminosarum*, the glucose-methanol-choline oxidoreductase GmcA, symbiotic nitrogen fixation, antioxidant and symbiotic gene expression, quantitative proteomics

## Abstract

GmcA is a FAD-containing enzyme belonging to the GMC (glucose-methanol-choline oxidase) family of oxidoreductases. A mutation in the *Rhizobium leguminosarum gmcA* gene was generated by homologous recombination. The mutation in *gmcA* did not affect the growth of *R. leguminosarum*, but it displayed decreased antioxidative capacity at H_2_O_2_ conditions higher than 5 mM. The *gmcA* mutant strain displayed no difference of glutathione reductase activity, but significantly lower level of the glutathione peroxidase activity than the wild type. Although the *gmcA* mutant was able to induce the formation of nodules, the symbiotic ability was severely impaired, which led to an abnormal nodulation phenotype coupled to a 30% reduction in the nitrogen fixation capacity. The observation on ultrastructure of 4-week pea nodules showed that the mutant bacteroids tended to start senescence earlier and accumulate poly-β-hydroxybutyrate (PHB) granules. In addition, the *gmcA* mutant was severely impaired in rhizosphere colonization. Real-time quantitative PCR showed that the *gmcA* gene expression was significantly up-regulated in all the detected stages of nodule development, and statistically significant decreases in the expression of the redoxin genes *katG, katE*, and *ohrB* were found in *gmcA* mutant bacteroids. LC-MS/MS analysis quantitative proteomics techniques were employed to compare differential *gmcA* mutant root bacteroids in response to the wild type infection. Sixty differentially expressed proteins were identified including 33 up-regulated and 27 down-regulated proteins. By sorting the identified proteins according to metabolic function, 15 proteins were transporter protein, 12 proteins were related to stress response and virulence, and 9 proteins were related to transcription factor activity. Moreover, nine proteins related to amino acid metabolism were over-expressed.

## Introduction

*Rhizobium leguminosarum* bv. *viciae* is an aerobic, Gram-negative, nitrogen-fixing bacterium that can live under the conditions of microaerobe, aerobe and form symbiotic relationships with *Pisum sativum* (pea) and *Vicia cracca* (vetch) under the condition of nitrogen limitation (Karunakaran et al., 2009). Organisms of this genus play a critical role in soil fertility, inducing the formation of symbiotic nodules on the roots of leguminous plants, where bacteroids reduce atmospheric nitrogen to ammonia available for plant uptake (Bhat et al., 2015). The symbiosis between rhizobia and legumes can be characterized by a mutual exchange of signal molecules between the two partners (Janczarek et al., 2015; López-Baena et al., 2016). After attachment of the bacteria to the plant root, the plant supports bacterial infection via host-derived infection threads (Haney and Long, 2010). Successful nodulation requires the activation of cell division in the cortex to form the nodule primordium (Blanco et al., 2009). In nodules, the nitrogenase enzyme, which is extremely sensitive to oxygen, has a low turnover number and a large requirement of chemical energy in the form of ATP and reducing potential (Clarke et al., 2011; Okazaki et al., 2015). In addition to reducing N_2_ and protons, nitrogenase can also reduce several small, non-physiological substrates, including a wide array of carbon-containing compounds (Seefeldt et al., 2013). It was found that uptake hydrogenases allow rhizobia to recycle the hydrogen generated in the nitrogen fixation process within the legume nodule (Baginsky et al., 2002).

Oxidoreductases catalyze a large variety of specific reduction, oxidation, and oxyfunctionalization reactions, which are important in redox processes, transferring electrons from a reductant to an oxidant (Hollmann and Schmid, 2004; Jeelani et al., 2010). Oxidoreductases included laccases, GMC (glucose-methanol-choline) oxidoreductases, copper radical oxidases and catalases (Beckett et al., 2015). The family of GMC flavoprotein oxidoreductases, which includes glucose/alcohol oxidase and glucose/choline dehydrogenase from prokaryotic and eukaryotic organisms, was first outlined by Cavener (1992). Members of the GMC oxidoreductase family share a common structural backbone of an adenine-dinucleotide-phosphate-binding βαβ-fold close to their amino terminus (Iida et al., 2007). The group of GMC flavoprotein oxidoreductases encompasses glucose oxidase from the mold *Aspergillus niger*, the glucose dehydrogenase from *Thermoplasma acidophilum* and *Drosophila melanogaster*, methanol oxidase from yeast *Hansenula polymorpha*, and choline dehydrogenase from *Escherichia coli* (Ahmad et al., 2010; Liu et al., 2013). In the leaf beetle subtribe *Chrysomelina sensu stricto*, GMC oxidoreductases enabled chemical defenses and were important for adaptive processes in plant–insect interactions (Rahfeld et al., 2014). In *E. coli*, choline dehydrogenase catalyzes the flavin-dependent, two-step oxidation of choline to glycine betaine, which acts as an osmoprotectant compatible solute that accumulates when the cells are exposed to drastic environmental changes in osmolarity (Yilmaz and Bülow, 2010). However, little is known about the functional diversity of the rhizobium GMC family.

*Rhizobium leguminosarum* bv. *viciae*, which has been widely used as a model to study nodule biochemistry, is able to nodulate and fix nitrogen in symbiosis with several legumes (Karunakaran et al., 2009). Here, we investigated the roles of a GMC oxidoreductase GmcA in free-living bacteria and during nitrogen-fixing symbiosis on pea by analyzing the phenotypes of a mutant strain. Proteome analysis provides clues to explain the differences between the *gmcA* mutant and wild-type nodules.

## Materials and Methods

### Bacterial Growth and Media

The strains, plasmids and primers used in this study are listed in Table 1. *Rhizobium* strains were grown at 28°C in either Tryptone Yeast extract (TY) (Beringer and Hopwood, 1976) or Acid Minimal Salts medium (AMS) (Poole et al., 1994) with D-glucose (10 mM) as a carbon source and NH_4_Cl (10 mM) as a N source (referred to as AMS Glc/NH_4_^+^). For growth and qRT-PCR experiments, cells were grown in AMS Glc/NH_4_^+^. Antibiotics were used at the following concentrations (μg/mL): ampicillin (Amp), 50; gentamicin (Gm), 20; kanamycin (Km), 20; neomycin (Neo), 80; spectinomycin (Spe), 100; streptomycin (Str), 500; tetracycline (Tc), 5. Strains were grown at 28°C with shaking (200 rpm) for liquid media. To monitor culture growth, optical density at 600 nm (OD_600_) was measured on three independent cultures.

**TABLE 1:** Strains, plasmids, and primers.

**Strains**	**Description**	
RL3841	*Rhizobium leguminosarum* bv. *viciae*, Str^r^	
RLgmcA	Rl3841 *gmcA*:pk19mob, Str^r^ Neo^r^	
RLgmcA (pBBRgmcA)	RLgmcA carrying *gmcA* gene, Str^r^ Neo^r^ Gm^r^	
Plasmids	Description	
pK19mob	pK19mob pUC19 derivative *lacZ mob*; Km^r^	
pRK2013	Helper plasmid for mobilizing plasmids; Km^r^	
pKgmcA	gmcAF/gmcAR PCR product in pK19mob, Km^r^	
pBBRgmcA	cgmcAF/cgmcAR PCR product in pBBR1MCS-5, Gm^r^	
**Primer**	**Description**	**Sequence^1^**
gmcAF	Sense primer for pRL100444 (*gmcA*) mutation	TTTAGATCTGGCGGGTTCCTTTGCGGTAA
gmcAR	Antisense prime for pRL100444 (*gmcA*) mutation	TTTCTGCAGTCAGCTCACCGGTCGCCTTT
MgmcA	Mapping PCR primer for *gmcA* gene	CGCCCGACGGATTGTAGAAT
pK19A	pK19mob mapping primer	ATCAGATCTTGATCCCCTGC
pK19B	pK19mob mapping primer	GCACGAGGGAGCTTCCAGGG
gyrB1-F	Sense primer for qRT-PCR of *GyrB1*	GGCATCACCAAAAGGGAAAA
gyrB1-R	Antisense primer for qRT-PCR of *GyrB1*	GCGAGGAGAATTTCGGATCA
cgmcAF	Sense primer for *gmcA* complementation	TTTGGTACCAGCTCACTGTCGATCTCTCC
cgmcAR	Antisense prime for *gmcA* complementation	TTTTCTAGACCTTTATCCGGTTGAGCTGG
QgmcA -for	Sense primer for qRT-PCR of *gmcA*	CGCCGCCTCGCTCGGCAAGA
QgmcA-rev	Antisense primer for qRT-PCR of *gmcA*	ATGCTCATGGAACTGCGAAG
*gyrB1*-F	*gyrB1* primers for qRT-PCR	GGCATCACCAAAAGGGAAAA
*gyrB1*-R		GCGAGGAGAATTTCGGATCA
QkatG-F	*katG* primers for qRT-PCR	GCAACTATTACGTCGGTCTG
QkatG-R		TCTCATCGATGACATTTTCC
QkatE-F	*katE* primers for qRT-PCR	CTCTCATCGATGACTTCCAT
QkatE-R		GGGACTCATATGTTTCGAAG
Q*orhB*_F	Sense primer for qRT-PCR of *orhB*	CGGGCAGGCTGACATTGAGG
Q*orhB*_R	Antisense primer for qRT-PCR of *orhB*	GCTGCTCAGAGAAAGATCAC
QhmuS-F	*hmuS* primers for qRT-PCR	AAGACCAGTCGCAGGAATTT
QhmuS-R		GAAGAACTCATGCGTATCGG
Q*nifD*_F	Sense primer for qRT-PCR of *nifD*	GCAACTATTACGTCGGTCTG
Q*nifD*_R	Antisense primer for qRT-PCR of *nifD*	TCTCATCGATGACATTTTCC
Q*fdxB*_F	Sense primer for qRT-PCR of *fdxB*	ATGGCGAAGACGACTTTAAT
Q*fdxB*_R	Antisense primer for qRT-PCR of *fdxB*	ATGAGTCTGGCAGTTCTTGG

*^1^Restriction sites in primer sequences are underlined.*

### Construction and Complementation of the *gmcA* Gene Mutant of *R. leguminosarum* 3841

Primers gmcAF and gmcAR were used to PCR amplify an internal region of the *gmcA* gene from *R. leguminosarum* bv. *viciae* 3841 genomic DNA (Johnston and Beringer, 1975). The 650-bp *gmcA* PCR product was cloned into the *Pst*I and *Xba*I sites of pK19mob, resulting in plasmid pKgmcA. The plasmid pKgmcA was conjugated with *R. leguminosarum* bv. *viciae* 3841 using pRK2013 as a helper plasmid, as previously described (Figurski and Helinski, 1979; Karunakaran et al., 2010). Insertions into the *gmcA* gene of strain RL3841 were selected by neomycin resistant AMS medium with 30mM pyruvate as a sole carbon source and confirmed by PCR using MgmcA and a pK19mob-specific primer (either pK19A or pK19B) (Karunakaran et al., 2010).

To complement the *gmcA* mutant, primers cgmcAF and cgmcAR were used to amplify the complete *gmcA* gene from strain RL3841. The PCR product was digested with *Kpn*I and *Xba*I and cloned into pBBR1MCS-5, resulting in plasmid pBBRgmcA. Plasmid pBBRgmcA was conjugated into the mutant strain RLgmcA using pRK2013 as a helper plasmid to provide the transfer genes, as previously described (Karunakaran et al., 2010).

### Hydrogen Peroxide Resistance Activity

Logarithmic phase cultures of mutant strain RLgmcA and wild-type RL3841 were collected and washed twice in sterile phosphate-buffered saline (PBS) (1×; 136 mM NaCl, 2.6 mM KCl, 8.0 mM Na_2_HPO_4_, and 1.5 mM KH_2_PO_4_). Cells with an optical density (OD_600_) 1 were treated with H_2_O_2_ at different concentrations (0, 1, 5, and 10 mmol/L) for 1 h. Strains were thoroughly washed with distilled water to remove any remaining oxidant, and the diluted TY plate method was used to evaluate the bacterial survival rate. The experiment consisted of three independent experiments, each of which had three repeats, and statistical differences were analyzed with one-way ANOVA (*P* < 0.05).

### Enzyme Activity Experiments

For analysis of glutathione reductase and glutathione peroxidase activities, logarithmic phase cultures of mutant strain RLgmcA and wild-type RL3841 with an optical density (OD_600_) 1 were collected, and treated with 5 mM H_2_O_2_ for 1 h. H_2_O_2_-treated PBS cells were collected by centrifugation at 5,000 rpm for 5 min at 4°C. The cells were held in an ice-water bath and sonicated for 15 min. The sonicate was centrifuged at 12,000 rpm for 10 min at 4°C. Glutathione reductase and glutathione peroxidase activities were determined using a peroxidase assay kit (Beyotime, China). The experiment consisted of three independent experiments, each of which had three repeats, and statistical differences were analyzed with one-way ANOVA (*P* < 0.05).

### Plant Growth and Microscope Study of Nodules

Pea seeds were surface sterilized in 95% ethanol for 30 s and then immersed in a solution of 2% sodium hypochlorite for 10 min. *R. leguminosarum* bv. *viciae* strains were inoculated with 10^7^ CFU per seed at the time of sowing. Plants were incubated in a controlled-environment chamber with an 18-h photoperiod (day/night temperature, 22 and 20°C). For dry weight determination, plants were grown in a 2-L beaker filled with sterile vermiculite, watered with nitrogen-free nutrient solution and harvested at 7 weeks (Cheng et al., 2017). The shoot was removed from the root and dried at 70°C in a dry-heat incubator for 3 days before being weighed. Acetylene reduction was determined at flowering (4 weeks) in peas, as previously described (Allaway et al., 2000). The experiment consisted of two independent experiments, each of which had five repeats, and statistical differences were analyzed with one-way ANOVA (*P* < 0.05).

Nodules at 4 weeks post infection were fixed in 2.5% glutaraldehyde and postfixed in 1.5% osmium tetroxide. Root nodules were sectioned and were then stained with toluidine blue. Ultra-thin sections stained with uranyl acetate and lead citrate were observed using a Hitachi H-7100 transmission electron microscope (Yan et al., 2004). For light microscopy, thick sections were cut on a microtome and stained.

### Rhizosphere Colonization

Rhizosphere colonization assays were performed as previously described (Cheng et al., 2017). Pea seedlings were grown for 7 days, as described above, for acetylene reduction, and inoculated with RLgmcA and RL3841 in the cfu ratios 1000:0, 0:1000, 1000:1000, and 10000:1000. After 7 days (14 days after sowing), shoots were cut-off and 20 mL of sterile phosphate-buffered saline (PBS) was added to the roots and vortexed for 15 min at speed 10 (Karunakaran et al., 2006). After vortexing, the samples were serially diluted and plate counted on TY medium plates containing either streptomycin (for wild-type RL3841 and mutant RLgmcA together) or streptomycin and neomycin (for RLgmcA), giving the total number of viable rhizosphere- and root-associated bacteria (Barr et al., 2008). Each treatment consisted of 10 replications, and statistical differences were analyzed with one-way ANOVA (*P* < 0.05).

### RNA Isolation and Quantitative Reverse Transcription–PCR (RT-PCR)

Quantitative Real-Time RT-PCR was used to determine differences in the expression of genes. Cell samples were collected from free-living *R. leguminosarum* cultivated in AMS liquid medium, or free-living cells treated with 5 mM H_2_O_2_ for 1 h or root nodules, which were harvested from pea that had been inoculated with *R. leguminosarum* strains at 2, 4, and 6 weeks. The nodules of plants were harvested and grinded into a regular fine powder with liquid nitrogen. Total RNA of each sample was extracted using TRIzol Reagent (Invitrogen) and quantified by NanoDrop (Thermo Fisher Scientific) (Smith et al., 1985). cDNA was prepared using SuperScript^TM^ II reverse transcriptase and random hexamers. Quantitative real-time PCR was performed using the SYBR Premix ExTaq (Takara, Dalian, China) on the BIO-RAD CFX96 Real-Time PCR Detection System. Primers for *katG, katE, hmuS, ohrB, rhtA*, and *nifD* are detailed in Table 1. *Gyrb1* was used as a reference housekeeping gene and the obtained data were analyzed as previously described (Prell et al., 2009). Statistical analysis of data sets was performed using REST (Pfaffl et al., 2002).

### Protein Extraction and LC-MS/MS Analysis

The 4-week-nodule samples were grinded into cell powder in liquid nitrogen. The cell powder was transferred to a 5-mL centrifuge tube. Four volumes of lysis buffer (8 M urea, 1% protease inhibitor cocktail) was then added to the cell powder, and the slurry was sonicated three times on ice using a high intensity ultrasonic processor. Cellular debris was removed by centrifugation at 12,000 *g* for 10 min at 4°C, the supernatant was collected, and the protein content was determined using BCA protein assay kit (Pierce, Rockland, IL, United States). The resulting proteins were reduced by 5 mM dithiothreitol at 56°C for 30 min, and then alkylated in 11 mM iodoacetamide for 15 min at room temperature in the dark. Each protein sample was then diluted by 100 mM tetraethyl ammonium bromide (TEAB) to obtain a urea concentration of less than 2 and 1:100 trypsin-to-protein mass ratio for a second digestion of 4 h. Following trypsin digestion, the peptides then were desalted by Strata X C18 SPE column and vacuum-dried. The peptides were reconstituted in 0.5 M TEAB and processed according to the manufacturer’s instructions of the tandem mass tag (TMT) kit (Thermo Fisher Scientific, Bremen, United States). Concisely, one unit of TMT reagent was dissolved and reconstituted in acetonitrile. The peptide mixtures were then incubated at room temperature for 2 h and pooled, desalted and dried by vacuum centrifugation.

The tryptic peptides were dissolved in solvent A (0.1% formic acid in aqueous solution) and loaded directly onto a reversed phase analytical column (75 μm i.d. × 15 cm length). The loaded material was eluted from this column in a linear gradient of 6–22% solvent B (0.1% formic acid in 98% acetonitrile) over 26 min, 23–35% in 8 min, climbing to 80% in 3 min and holding at 80% for 3 min with a flow rate of 400 nL/min. The MS proteomics data were deposited to NSI source, followed by tandem mass spectrometry (MS/MS) by using a Q Exactive^TM^ Plus (Thermo) coupled online to the ultra-performance liquid chromatography (UPLC). The electrospray voltage was set to 2.0 kV. For the full scan mode, the m/z scan range was from 350 to 1,800. The intact peptides were detected in the Orbitrap at a resolution of 70,000. Peptides were selected to run MS/MS analysis using NCE setting as 28 and the fragments were measured using a resolution of 17,500 in the Orbitrap. The MS analysis alternated between MS and data-dependent tandem MS scans with 15.0 s dynamic exclusion. Automatic gain control (AGC) was set to accumulate 5 × 10^4^ ions with the Fixed first mass of 100 m/z. Experiments were conducted in triplicate.

### Data Analysis

The resulting MS/MS data were processed and prepared for a database search using the MaxQuant version 1.5.2.8 (Cox and Mann, 2008). The resulting tandem mass spectra were searched against the *R. leguminosarum* genome database concatenated with a reverse decoy database (Young et al., 2006). Trypsin/P was specified as the cleavage enzyme allowing up to four missing cleavages. The precursor mass tolerance was set to 20 ppm for the first search and 5 ppm for the main search, and the tolerance of the ions was set to 0.02 Da for fragment ion matches. Carbamidomethylation of cysteines was considered as a fixed modification, and oxidation of methionine was specified as variable modifications. A false discovery rate (FDR) of 1% was specified, and the minimal peptide score for modified peptides was set to 40. Protein expression was analyzed statistically using Student’s *t-*tests (*p* < 0.05). Up-regulated and down-regulated proteins were defined as having fold changes (FC) > 1.2 and <0.83, respectively.

## Results

### Bioinformatic Analysis of the *R. leguminosarum gmcA* Gene

*Rhizobium leguminosarum gmcA* gene (*pRL100444)* is predicted to encode a 550-amino acid polypeptide with an expected molecular mass of 60.5 kDa and a pI value of 8.19 (Young et al., 2006). The amino acid sequence of GmcA contained a consensus motif of a FAD/NAD(P)-binding domain in its N-terminal part and two GMC oxidoreductase signature patterns (Supplementary Figure S1), suggesting that GmcA should be included into the glucose-methanol-choline (GMC) flavin-dependent oxidoreductase family.

### Antioxidation Analysis of a *R. leguminosarum gmcA* Mutant

To confirm the function of the *gmcA* gene in growth performance, antioxidation and symbiotic nitrogen fixation ability, a mutant RLgmcA strain of this gene was constructed by single crossover homologous recombination. In liquid AMS minimal medium with glucose as a carbon source and NH_4_Cl as a nitrogen source, there is no significant difference in growth between the mutant RLgmcA and wild-type RL3841 (data not shown).

The importance of GmcA for protection against oxidative stress was investigated by carrying out survival assays of the mutant RLgmcA in the presence of oxide hydrogen peroxide (H_2_O_2_). The survival rates of RLgmcA were not significantly affected by H_2_O_2_ treatments at low concentrations of 0.5 and 1 mmol/L compared with the wild-type RL3841 strain, whereas the antioxidative capacity of mutant RLgmcA was significantly decreased by these treatments with H_2_O_2_ at higher concentrations of 5 and 10 mmol/L (Table 2). The role of *R. leguminosarum* GmcA in controlling protein glutathionylation status was investigated by quantifying glutathione reductase and glutathione peroxidase activities in 5 mM H_2_O_2_-induced oxidative stress conditions. The results showed that the glutathione reductase activity of mutant RLgmcA was not different from that of wild-type strain RL3841, but its glutathione peroxidase activity was significantly lower (Table 3). Thus, GmcA may play important roles in oxidative stress resistance and cellular detoxification in *R. leguminosarum*.

**TABLE 2:** Tolerance of *R. leguminosarum* stains to different concentrations of H_2_O_2_.

**Strain**	**H_2_O_2_ (mM)**				
	**0**	**0.5**	**1.0**	**5.0**	**10.0**
RL3841	(6.72 ± 0.71) × 10^8 a^	(4.27 ± 0.27) × 10^8 a^	(3.85 ± 0.18) × 10^8 a^	(2.73 ± 0.14) × 10^7 a^	(1.01 ± 0.21) × 10^7 a^
RLgmcA	(7.03 ± 1.00) × 10^8 a^	(3.80 ± 0.17) × 10^8 a^	(3.21 ± 0.29) × 10^8 a^	(1.59 ± 0.19) × 10^7b^	(4.07 ± 0.90) × 10^6b^

*All data are averages (±SEM) from three independent experiments. ^*a,b*^Different letters indicates the value is significantly different from that of the wild-type RL3841 control (one-way ANOVA, *P* < 0.05).*

**TABLE 3:** Oxidase activity of *R. leguminosarum gmcA* mutant.

**Strains *R. leguminosarum***	**Glutathione reductase (U/mg protein)**	**Glutathione peroxidase (U/mg protein)**
RL3841	0.399 ± 0.041^a^	0.406 ± 0.029^a^
RLgmcA	0.408 ± 0.031^a^	0.018 ± 0.006^b^
RLgmcA(pBBRgmcA)	0.409 ± 0.030^a^	0.385 ± 0.031^a^

*All data are averages (±SEM) from three independent experiments. ^*a,b*^Different letters indicates the value is significantly different from that of the wild-type RL3841 control (one-way ANOVA, *P* < 0.05).*

### Pea Rhizosphere Colonization by *R. leguminosarum* Strains

**FIGURE 1:**
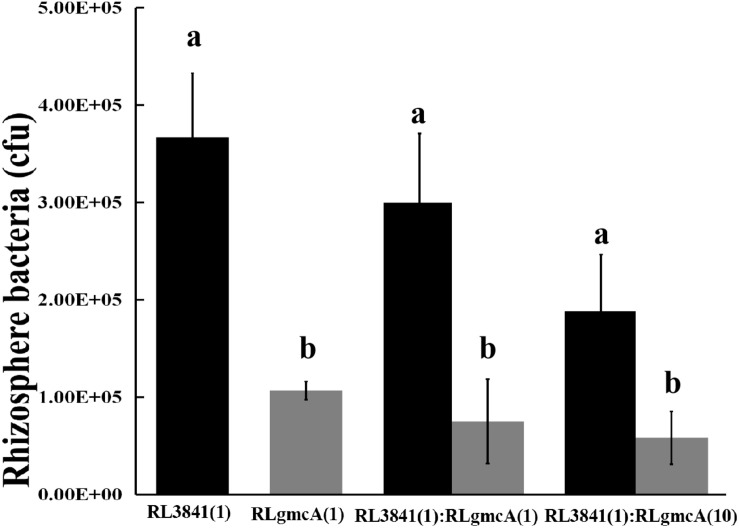
Competition of the wild type (RL3841) (black bars) and the *gmcA* mutant (RLgmcA) (gray bars) in sterile rhizospheres. Inoculation ratios are given on the *x-*axis, with 1 corresponding to 1,000 CFU. Number of bacteria (per plant) recovered from 10 plants (mean ± SEM) are shown. ^a,b^Different letters indicate the value is significantly different between mutant RLgmcA and wild-type RL3841 control (one-way ANOVA, *P* < 0.05).

Competition between the *gmcA* mutant RLgmcA and the wild type RL3841 for growth in the pea rhizosphere was measured by inoculating a low number of bacteria into the pea rhizosphere (10^3^ to 10^4^ bacteria per seedling) and determining total bacteria after 7 days. When the mutant RLgmcA and the wild type RL3841 were inoculated alone into short-term colonization of sterile pea rhizosphere, the percentage of bacteria recovered after 7 days was significantly lower for the mutant than for the wt strain (Figure 1). When inoculated in equal ratios, RLgmcA accounted for only 25% of bacteria recovered (*t-*test; *P* ≤ 0.01). Even when strain RLgmcA was inoculated at a 10-fold excess over the wild type, it still accounted for only 41% of bacteria recovered (Figure 1). The decreased ability of the *gmcA* mutant to grow in a sterile rhizosphere of peas shows that GmcA is essential for colonization of the pea rhizosphere by *R. leguminosarum*.

### The Symbiotic Phenotype of *R. leguminosarum* Strains

To observe the nodulation status and measure nitrogenase activity of the *gmcA* mutant strain, pea seedlings were inoculated with the mutant RLgmcA or wild-type RL3841. Four weeks later, the number, shape and structure, and acetylene reduction activity (ARA) values of the nodules were measured. No statistically significant difference was observed in the number of nodules per plant between plants inoculated strain RLgmcA and plants inoculated with wild-type RL3841 (Table 4 and Supplementary Figure S2). *R. leguminosarum* bv. *viciae* formed determinate nodules on pea, while the *gmcA* mutant elicited more elongated, rather than spherical, nodules compared to the wild type and showed a 30.36% decrease in ARA and a 40% drop in the dry weight of plants compared to the wild type (Table 4). When recombinant plasmid pBBRgmcA was introduced into mutant RLgmcA, plants inoculated with the resulting strain RLgmcA(pBBRgmcA) formed normal nodules and showed no significant difference in nitrogen-fixing ability and the dry weight of plants compared to the RL3841-inoculated plants (Table 4).

**TABLE 4:** Symbiotic behavior of *R. leguminosarum gmcA* mutant.

Strain	Nodules per plant	Acetylene reduction (μ moles acetylene per plant per h)	Dry weight per plant (g)
RL3841	137.3 ± 13.2^a^	2.24 ± 0.14^a^	1.86 ± 0.20^a^
RLgmcA	131.3 ± 11.3^a^	1.56 ± 0.05^b^	1.10 ± 0.18^b^
RLgmcA(pBBRgmcA)	135.5 ± 11.3^a^	2.12 ± 0.16^a^	1.80 ± 0.16^a^
WC	0	0	0.35 ± 0.09^c^

*All data are averages (±SEM) from ten independent plants. ^*a,b,c*^Different letters indicates the value is significantly different from that of the wild-type RL3841 control (one-way ANOVA, *P* < 0.05). WC, water control without inoculation.*

**FIGURE 2:**
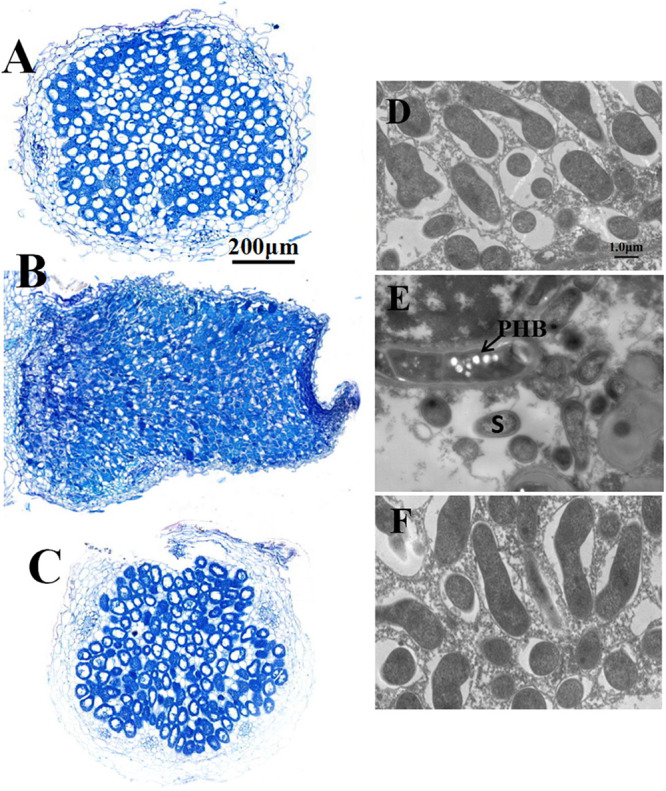
Structure of 4-week-old pea nodules and bacteroids. Nodules were induced by RLgmcA(pBBRgmcA) **(A,D)**, RLgmcA **(B,E)**, RL3841 **(C,F)**. The wild-type RL3841 forms normal spherical (determinant) nodules **(C)**, while the *gmcA* mutant forms elongated nodules **(B)**. PHB, poly-β-hydroxybutyrate. S, senescing bacteroid. Scale bars = 200 μm **(A–C)** and 1 μm **(D–F)**.

Four-week-old nodules were further examined by both light and electron microscopy. The nodules induced both by wild type RL3841 and by mutant RLgmcA turned blue when stained with toluidine blue. These observations were corroborated by light microscopic analysis. Both the nodules were filled by Rhizobia-infected cells (Figures 2B,C). The ultrastructural structure of the infected cells was observed by transmission electron microscopy. In the mutant infected nodule cells, bacteroids underwent premature senescence. Bacteroids in pea plants inoculated by *R. leguminosarum* bv. *viciae* usually did not produce visible PHB granules, but in the mutant bacteroids, the poly-b-hydroxybutyrate (PHB) was also distinctly observed (Figure 2).

### Expression Level of the *gmcA* Gene in Nodules Induced by *R. leguminosarum* 3841

**FIGURE 3:**
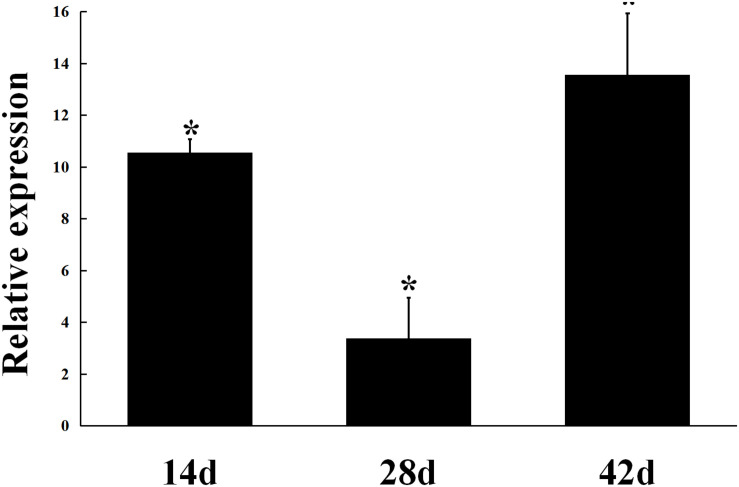
Expression patterns of *gmcA* gene in symbiotic nodules. Gene expression levels were examined by real-time RT-PCR. Nodules were collected on different days after inoculation with RL3841. Relative expression of *gmcA* gene in symbiotic nodule bacteroids compared with RL3841 wild type cells growth in AMS Glc/NH_4_^+^ medium. Data are the average of three independent biological samples (each with three technical replicates). Superscript asterisk indicates significant difference in relative expression (>2-fold, *P* ≤ 0.05).

The expression of *gmcA* was significantly up-regulated in the early stage (14 days), maturation stage (28 days) and late stage (42 days) of nodule development and senescence in comparison to that in free-living cells (Figure 3). During symbiosis, *gmcA* gene has the highest expression level in nodules at 42 days after inoculation. Thus, these results showed that *gmcA* gene expression was induced during *R. leguminosarum*-pea symbiosis and suggest that this gene plays an important role in bacteroid persistence in old nodules.

### Analysis of the Relative Expression of Genes Involved in Redoxin Production and Nitrogen Fixation in the *gmcA* Mutant

**FIGURE 4:**
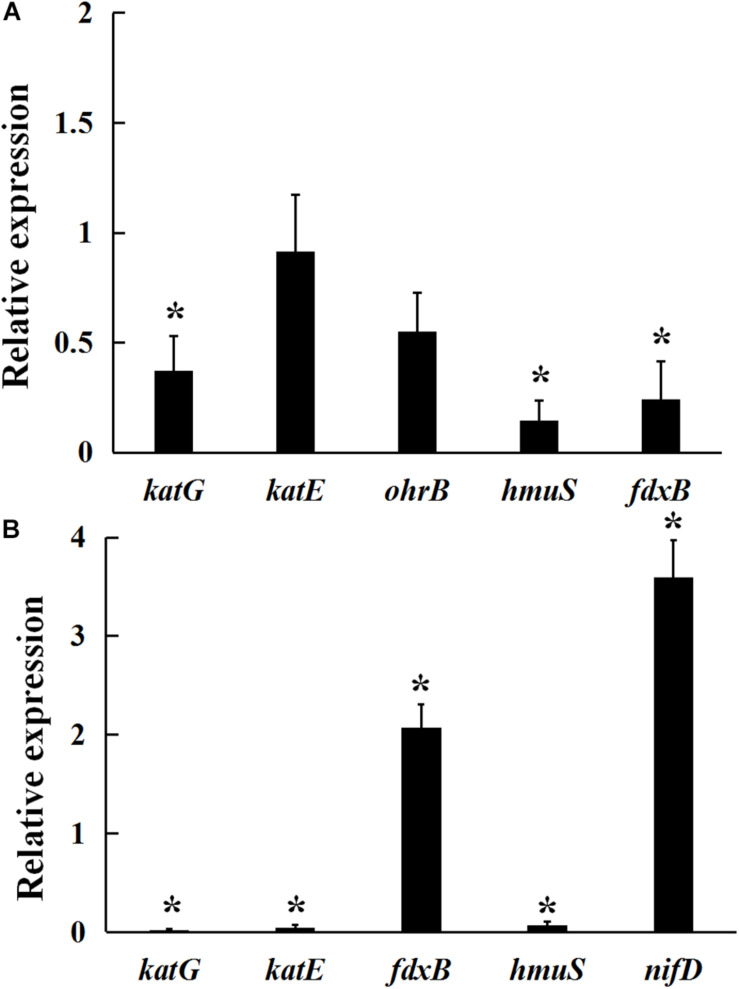
Relative expression of genes involved in hydrogen peroxide stresses **(A)** and 4-week-nodule bacteroids **(B)** in *gmcA* mutant compared with wild-type RL3841 measured by qRT-PCR. The value of relative gene expression in wild-type strain **(A)** or bacteroid **(B)** is given to 1.0, and the ratio was the expression level of each gene in mutant RLgmcA vs. in wild-type RL3841. Data are the average of three independent biological samples (each with three technical replicates). Superscript asterisk indicates significant difference in relative expression (>2-fold for up-expression or <2-fold for down-expression, *P* ≤ 0.05).

As shown in Figure 4A, under 5 mmol/L H_2_O_2_-induced oxidative stress condition, a significant decrease in *katG, fdxB*, and *hmuS* gene expression was observed in the *gmcA* mutant, suggesting that GmcA plays an important role in cellular redox balance. Since a large reduction in the nitrogen-fixing capacity of nodules inoculated with mutant strain was observed, qRT-PCR was used to assess whether the N-fixation system, e.g., nitrogenase genes, was affected in the transcription of ribosomal RNAs in the GmcA-deficient mutant. The expression of *nifD* and *fdxB* was analyzed in pea root nodules using qRT-PCR (Figure 4B). Unexpectedly, the expression level of *nifD* and fdxB was found to be significantly increased in 4-week-old nodules inoculated with *gmcA* mutant strain compared with control nodules. Thus, GmcA may function in redox balance and antioxidant defense system in the pea root nodules. *hmuS* gene expression was significantly down-regulated in *gmcA* mutant, both under H_2_O_2_-induced oxidative stress condition and in 4-week-old nodule, suggesting *gmcA* is involved in iron transport and regulation of iron homeostasis.

### Protein Differential Expression Analysis

A quantitative proteomic approach using UPLC coupled with tandem mass spectrometry (LC/LC-MS/MS) was performed to compare differential *gmcA* mutant root bacteroids in response to wild type infection. Proteomics analysis identified peptides derived from a total of 2002 distinct protein groups in *gmcA* mutant bacteroids and 2000 in wild-type bacteroids, with molecular weights ranging from 7 to 317 kDa. A total of 60 differentially expressed proteins (*P* < 0.05) were identified. Among these proteins (Table 5), 33 proteins were up-regulated in *gmcA* mutant nodule bacteroids and 27 proteins were down-regulated. Cell surface protein (RL4381) was absent in the *gmcA* mutant bacteroids, while invasion associated protein (RL1020) and lipoate-protein ligase B (RL2555) were not found in the wild-type bacteroids. Thirty-two differential protein-encoding genes were localized in plasmids pRL7, pRL8, pRL9, pRL10, pRL11, and pRL12. Cellular localization of the differentially expressed proteins showed that thirty-nine proteins localized to the cytoplasm, thirteen proteins localized to periplasmic space, five proteins located in the outer membrane, two were extracellular proteins, and one protein existed in the inner membrane (Table 5).

**TABLE 5:** Differential expression proteins in 4-week nodule mutant bacteroids relative to wild-type bacteroids.

**Gene ID**	**Gene Name**	**Cellular localization**	**Protein description**	**MW [kDa]**	**pI**	**Ratio**	***P*-value**
**Stress response and virulence**							
RL3853		Cytoplasmic	FAD-dependent oxidoreductase	47.38	5.71	6.15	0.0004
RL4381		Outermembrane	cell surface protein	66.06	4.46	1.58	NP1
pRL90097	*pdxA2*	Cytoplasmic	4-hydroxythreonine-4-phosphate dehydrogenase	34.66	6.39	1.23	0.0332
pRL100245		Cytoplasmic	LLM class flavin-dependent oxidoreductase	38.97	5.20	1.22	0.0082
pRL80022		Cytoplasmic	alpha/beta hydrolase	35.12	6.03	−0.40	0.0011
pRL80023	*cutM*	Cytoplasmic	carbon monoxide dehydrogenase subunit M protein	30.42	5.56	−0.48	0.0012
pRL80041	*hisD*	Cytoplasmic	Histidinol dehydrogenase	47.16	5.39	−0.60	0.0115
RL1020		Periplasmic	invasion associated protein	22.08	5.45	−0.85	NP2
pRL120603	*gabD3*	Cytoplasmic	NAD-dependent succinate-semialdehyde dehydrogenase	52.53	5.29	−0.74	0.0344
pRL90027	*adhA*	Cytoplasmic	alcohol dehydrogenase	37.15	5.93	−0.77	0.0008
pRL120056	*mcpR*	Cytoplasmic	methyl-accepting chemotaxis protein	68.73	5.01	−0.77	0.0426
pRL90018	*fixN2*	Innermembrane	Putative cytochrome oxidase transmembrane component FixN	60.90	8.98	−0.82	0.0074
**Amino acid metabolism**							
pRL100242		Cytoplasmic	amino acid synthesis family protein	21.17	6.29	1.45	0.0159
pRL110557	*glxB*	Cytoplasmic	glutamine amidotransferase	31.99	5.21	1.42	0.0190
RL0041	*hisE*	Cytoplasmic	Phosphoribosyl-ATP pyrophosphatase	11.51	5.19	1.30	0.0496
pRL100099		Cytoplasmic	Nif11 family protein	14.45	8.86	1.23	0.0152
RL2075	*gatC*	Cytoplasmic	Aspartyl/glutamyl-tRNA(Asn/Gln) amidotransferase subunit C	10.19	4.73	1.22	0.0082
pRL100192		Outermembrane	glutamate N-acetyltransferase	20.33	6.71	1.21	0.0024
pRL90221		Cytoplasmic	Putative glutamine amidotransferase protein	28.31	5.18	−0.72	0.0244
**Carbohydrate metabolism**							
pRL110453		Cytoplasmic	Concanavalin A-like lectin/glucanase domain	22.05	5.00	1.25	0.0237
RL0916	*dgoK*	Cytoplasmic	2-dehydro-3-deoxygalactonokinase	31.49	5.69	−0.73	0.0076
RL0874	*RL0874*	Cytoplasmic	aldo/keto reductase	38.21	5.45	−0.78	0.0000
pRL110598		Cytoplasmic	L-fuconate dehydratase	47.20	5.17	−0.82	0.0137
pRL120643	*groSp12*	Cytoplasmic	co-chaperone GroES	11.37	5.48	−0.83	0.0003
RL2555	*lipB*	Cytoplasmic	lipoate-protein ligase B	26.70	5.22	−0.99	NP2
**Transporter activity**							
RL4326		Periplasmic	Putative transmembrane protein	98.15	5.59	1.39	0.0122
RL3066		Periplasmic	Putative transmembrane protein	17.22	8.09	1.35	0.0090
RL2491		Cytoplasmic	Conserved hypothetical exported protein	9.75	5.38	1.28	0.0001
pRL100386		Periplasmic	VWA domain-containing protein	74.90	4.84	1.24	0.0122
RL3065		Periplasmic	Conserved hypothetical exported protein	14.77	5.88	1.23	0.0151
pRL70182		Periplasmic	Conserved hypothetical exported protein	36.84	5.32	1.21	0.0265
pRL80026	*livJ*	Periplasmic	ABC transporter substrate-binding protein	45.36	5.81	−0.33	0.0006
pRL80060		Periplasmic	ABC transporter substrate-binding protein	29.75	5.35	−0.53	0.0007
pRL80085		Cytoplasmic	Autoinducer 2 ABC transporter substrate-binding protein	35.70	6.02	−0.60	0.0448
RL2775	*ropA1*	Outermembrane	Porin	36.80	3.92	−0.71	0.0111
RL4402		Cytoplasmic	ABC transporter substrate-binding protein	35.94	4.96	−0.72	0.0115
RL1499	*ropA2*	Outermembrane	Porin	36.71	4.01	−0.77	0.0001
pRL100415		Periplasmic	ABC transporter substrate-binding protein	37.92	5.17	−0.79	0.0492
pRL120671		Periplasmic	nitrate ABC transporter substrate-binding protein	36.00	5.50	−0.83	0.0123
pRL100325	*fhuA1*	Outermembrane	outer membrane siderophore receptor	78.19	4.64	−0.83	0.0203
**Nucleotide metabolism**							
RL0952		Cytoplasmic	RNA-binding domain transcriptional regulator	83.38	6.67	1.34	0.0278
RL2475	*holB*	Cytoplasmic	Putative DNA polymerase III, delta subunit	36.35	5.90	1.26	0.0409
RL1785	*rplX*	Cytoplasmic	50S ribosomal protein L5	11.21	10.37	1.23	0.0038
RL2183		Cytoplasmic	nucleotidyltransferase	33.59	8.80	−0.82	0.017
**Transcription factor activity**							
pRL100146		Periplasmic	transcriptional regulator	23.94	9.57	1.33	0.0243
RL4412	*priA*	Cytoplasmic	primosome assembly protein PriA	80.82	6.32	1.30	0.0382
RL3457		Extracellular	SH3-like domain, bacterial-type; uncharacterized protein	21.84	4.62	1.25	0.0082
RL0425	*ptsN*	Cytoplasmic	PTS IIA-like nitrogen regulatory protein PtsN	16.65	5.70	1.23	0.0007
RL1379	*rosR*	Cytoplasmic	MucR family transcriptional regulator	15.62	6.96	1.22	0.0055
RL0133		Cytoplasmic	YbaB/EbfC family nucleoid-associated protein	11.42	5.18	1.22	0.0007
pRL100196	*nifA*	Cytoplasmic	nif-specific transcriptional activator	56.46	9.05	1.21	0.0011
pRL80079		Cytoplasmic	sugar-binding transcriptional regulator	35.34	5.71	−0.38	0.0072
pRL80046		Cytoplasmic	TetR/AcrR family transcriptional regulator	25.32	8.01	−0.46	0.0112
**Unknown function proteins**							
pRL100106		Periplasmic	Uncharacterized protein	28.65	6.19	1.59	0.0363
RL1874		Cytoplasmic	Uncharacterized protein	13.23	4.66	1.35	0.0185
RL3516		Extracellular	DUF2076 domain-containing protein	27.48	4.26	1.24	0.0017
RL4728		Periplasmic	DUF1013 domain-containing protein	26.14	5.91	1.23	0.0005
RL2820		Cytoplasmic	Uncharacterized protein	7.40	9.46	1.23	0.0494
pRL80010		Cytoplasmic	Uncharacterized protein	9.58	9.51	−0.21	0.0189
pRL80005		Cytoplasmic	Uncharacterized protein	59.58	5.52	−0.47	0.0028

*Protein expression was analyzed statistically using Student’s *t*-tests (*P* < 0.05). Np1, no protein in mutant; Np2, no protein detected in wild type.*

By sorting the identified proteins according to metabolic function, most of the differences in expression were found among transporter activity (15 proteins), followed by 12 proteins related to stress response and virulence, 9 proteins related to transcription factor activity, 7 proteins related to amino acid metabolism, 6 proteins related to carbohydrate metabolism, and 4 proteins related to nucleotide metabolism. This change in metabolism was mirrored by corresponding changes in proteins involved in the regulation of transcription, among which, a nif-specific transcriptional activator NifA and a nitrogen regulatory protein PtsN were highly expressed in the mutant bacteroids. The main groups of differentially expressed proteins identified were transport proteins, of which 6 were ABC-type nitrate/nitrite transporters. The result showed *gmcA* mutant was affected in transport, especially in nitrate transport. Further analysis of the differentially expressed proteins identified a subset involved in stress response and virulence. The number of affected oxidoreductases, cytochrome oxidase, dehydrogenase, hydrolase, dehydrogenase, surface, and invasion associated proteins also suggests that GmcA function in antioxidant capacity in the root nodules and that the loss of these proteins could result in antioxidant defect. Finally, the loss of GmcA resulted in the differential expression of seven proteins with unknown function in the nodule bacteroids.

## Discussion

The family of GMC oxidoreductases includes glucose/alcohol oxidase and glucose/choline dehydrogenase. Members of this family catalyze a wide variety of redox reactions with respect to substrates and co-substrates (Sützl et al., 2018). An important issue is that *gmcA* expression is elevated in nitrogen-fixing bacteroids of the pea root nodules, but the function of GmcA in root nodule bacteria nitrogen fixing system is poorly understood. In this study, we took advantage of a *gmcA* mutant strain of *R. leguminosarum* to examine what role GmcA may play in symbiotic nitrogen fixation. Our data demonstrated that GmcA is required for the nodule senescence and cellular detoxification that is affected, regarding its nitrogen fixation capacity and oxidative stress response.

Mutation of *R. leguminosarum gmcA* did not affect the growth of free-living bacteria but led to decreased antioxidative capacity under the conditions of 5 and 10 mM hydrogen peroxide H_2_O_2_. The direct link between GmcA and H_2_O_2_ detoxification has been less reported, while in most wood-rotting fungi, the members of GMC oxidoreductase superfamily play a central role in the degradation process because they generate extracellular H_2_O_2_, acting as the ultimate oxidizer (Ferreira et al., 2015). Our results suggested that cells with GmcA tolerate internally generated or exogenously applied H_2_O_2_. Cellular oxidoreductases catalyze redox processes by transferring electrons from a reductant to oxidant and are important for protection against oxidative stress (Bisogno et al., 2010). The ferredoxin-like protein (FdxB) and iron transport protein HmuS are ubiquitous electron transfer proteins participating in the iron-sulfur cluster biosynthesis and a wide variety of redox reactions (Chao et al., 2005; Gu et al., 2008). In *Rhizobium*, the peroxidases and the catalases KatG (catalase HPI), KatE (catalase), and OhrB (organic hydroperoxide resistance) were known to participate in the antioxidant defense mechanism against H_2_O_2_-induced stress (Vargas Mdel et al., 2003), and the two electron transfer proteins FdxB and HmuS are also involved in a wide variety of redox reactions (Chao et al., 2005; Gu et al., 2008). This cell cytotoxicity was relieved by inducing transcription of antioxidant genes (Jung and Kim, 2003). Expression levels of *katG, fdxB*, and *hmuS* genes were significantly down-regulated in the *gmcA* mutant under H_2_O_2_-induced oxidative stress. It has been reported that decreased ferredoxin-NADP(H) oxidoreductase (FNR) results in a more oxidized glutathione pool, while increasing FNR content results in a more reduced glutathione pool (Goss et al., 2012). Glutathione reductase activity in mutant RLgmcA was not different from that wild-type strain, but the absence of GmcA was associated with a 96.5% decrease in cellular glutathione peroxidase activity. Cellular peroxide deficit damages cellular macromolecules by reactive oxygen species (ROS), and glutathione peroxidases are one of the important ROS scavengers in the cell (Islam et al., 2015). The decrease of glutathione peroxidase activity is related to an uncontrolled increase of ROS (Giergiel et al., 2012).

Pea plants inoculated with the *gmcA* mutant exhibited a large decrease in the nitrogen-fixing activity of root nodules (reduced by more than 30%), although, the protein expression of NifA and PtsN was higher in the mutant bacteroids compared to that of wild type bacteroids. Two genes, *nifD* and *fdxB*, involved in metabolism related to nitrogen fixation and bacteroid maturation in pea root nodules (Capela et al., 2006) also had a higher level of expression in the mutant bacteroids. It has been reported that GMC oxidoreductases are involved in extracellular hydrogen peroxide and iron homeostasis (Rohr et al., 2013). Iron is required for symbiotic nitrogen fixation as a key component of multiple ferroproteins involved in this important biological process (Takanashi et al., 2013). *hmuS* was chosen based on previous studies, which showed that it was involved in iron transport (Chao et al., 2005). *hmuS* exhibited higher expression level in the mutant bacteroids, demonstrating the involvement of GMC in the regulation of iron homeostasis. Proteomic analysis of the mutant nodule bacteroids indicated that most of the differentially expressed proteins were involved in transporter activity, metabolism, and stress responses. These transporters may aid in regulation of ion and membrane potential homeostasis through their transport of nitrate, which is known to regulate the symbiosis (Vincill et al., 2005). These results indicated that GmcA is involved in a variety of metabolic processes, as has been described in *A. niger* and *E. coli* (Etxebeste et al., 2012; Liu et al., 2013).

The electron microscope investigation revealed that *gmcA* mutant altered the ultrastructure of pea nodules. GmcA can likely play a role in nodule senescence, since senescent parameters such as increased activities of enzymes of amino acid metabolism, PHB production, and an increase in the number of disintegrated bacteroids occurred. In addition, glutathione peroxidase activity dramatically decreased, and amino acid metabolism reflecting arginase activity was increased. *R. leguminosarum* bv. *viciae* forms determinate nodules on pea and usually does not produce visible PHB granules during symbiosis. PHB granules occurred in undergoing senescence bacteroids, which indicated that the energy and carbon metabolism has shifted (Xie et al., 2011). The PHB and tricarboxylic acid (TCA) cycles both start with acetyl-CoA. Under aerobic conditions, the TCA cycle is responsible for the complete oxidation of acetyl-CoA and formation of intermediates required for ATP production, but under oxygen limitation condition, when there is an inhibition of the TCA cycle by NADH or NADPH, the bacteroids favor PHB synthesis. During PHB synthesis, there is apparently a concomitant reduction in protein synthesis, a process coupled to ATP formation and utilization (Tal et al., 1990). In the symbiosis of the GmcA-deficiency mutant RLgmcA, the low expression of the catalase-peroxidase gene (*katG*), alpha/beta hydrolase (pRL80022), carbon monoxide dehydrogenase (pRL80023), succinate-semialdehyde dehydrogenase (pRL120603), and alcohol dehydrogenase (pRL90027) inhibited NAD(P)H oxidase activity. To allow continued operation of the TCA cycle, NAD(P)H was channeled into other biosynthesis reactions, such as PHB synthesis, for acting as reducing equivalents (Xie et al., 2011).

The *gmcA* gene expression is significantly up-regulated during the whole nodulation process, and its highest expression level occurred at 42 days after inoculation. Moreover, the *R. leguminosarum gmcA* mutant was unable to compete efficiently in the rhizosphere with its wild-type parent, which shows that bacterial GmcA is important for adaptation to the microenvironment of the plant host. Overall, considering the poor nitrogen-fixing ability of its nodules, the mutant in *gmcA* gene had a profound influence on the whole nodulation process.

## Data Availability Statement

The mass spectrometry proteomics data have been deposited to the ProteomeXchange Consortium via the PRIDE partner repository with the dataset identifier PXD017485.

## Author Contributions

GC conceived and designed the study. QZ, SL, and HW performed the experiments. GC, QZ, DH, and XL analyzed the results. GC and QZ wrote the manuscript. All authors read and approved the final manuscript.

## Conflict of Interest

The authors declare that the research was conducted in the absence of any commercial or financial relationships that could be construed as a potential conflict of interest.

## Supplementary Material

FIGURE S1 Analysis of the protein domains of GmcA in *Rhizobium leguminosarum* 3841. BetA, choline dehydrogenase or related flavoprotein; GMC_oxred_C, GMC oxidoreductase; GMC_mycofac_2, GMC family mycofactocin-associated oxidoreductase.

FIGURE S2 Plant growth test of the symbiotic ability of *R. leguminosarum*. **(A)** Control plant root inoculated with the wild type RL3841, **(B)** Plant root inoculated with RLgmcA.
