# Corrigendum: A GMC Oxidoreductase GmcA Is Required for Symbiotic Nitrogen Fixation in *Rhizobium leguminosarum* bv. *viciae*

**DOI:** 10.3389/fmicb.2021.774051

**Published:** 2021-12-15

**Authors:** Qian Zou, Sha Luo, Hetao Wu, Donglan He, Xiaohua Li, Guojun Cheng

**Affiliations:** Hubei Provincial Engineering and Technology Research Center for Resources and Utilization of Microbiology, College of Life Sciences, South-Central University for Nationalities, Wuhan, China

**Keywords:** *Rhizobium leguminosarum*, the glucose-methanol-choline oxidoreductase GmcA, symbiotic nitrogen fixation, antioxidant and symbiotic gene expression, quantitative proteomics

In the original article, there was a mistake in the legend for **Figure 2** as published. The part labels for nodules induced by RL3841 and RLgmcA(pBBRgmcA) were incorrect. The correct legend appears below:

“Structure of 4-week-old pea nodules and bacteroids. Nodules were induced by RLgmcA(pBBRgmcA) **(A,D)**, RLgmcA **(B,E)**, RL3841 **(C,F)**. The wild-type RL3841 forms normal spherical (determinant) nodules **(C)**, while the *gmcA* mutant forms elongated nodules **(B)**. PHB, poly-β-hydroxybutyrate. S, senescing bacteroid. Scale bars = 200 μm **(A–C)** and 1 μm **(D–F)**.”

In the original article, there was an error in the text. In the Results section, the citation for Figure 2 contained the wrong part labels. A correction has been made to **Results, The Symbiotic Phenotype of** ***R. leguminosarum* Strains**, paragraph 2:

“Four-week-old nodules were further examined by both light and electron microscopy. The nodules induced both by wild type RL3841 and by mutant RLgmcA turned blue when stained with toluidine blue. These observations were corroborated by light microscopic analysis. Both the nodules were filled by Rhizobia-infected cells (Figures 2B,C). The ultrastructural structure of the infected cells was observed by transmission electron microscopy. In the mutant infected nodule cells, bacteroids underwent premature senescence. Bacteroids in pea plants inoculated by *R. leguminosarum* bv. *viciae* usually did not produce visible PHB granules, but in the mutant bacteroids, the poly-b-hydroxybutyrate (PHB) was also distinctly observed (**Figure 2**).”

In the original article, there was a mistake in Table 4 as published. The strain names LMB599 and LMB675 were wrong; the correct strain names are RLgmcA and RLgmcA(pBBRgmcA), respectively. The corrected Table 4 appears below:

**Table 4:** Symbiotic behavior of *R. leguminosarum gmcA* mutant.

**Strain**	**Nodules per plant**	**Acetylene reduction (μ moles acetylene per plant per h)**	**Dry weight per plant (g)**
RL3841	137.3 ± 13.2^a^	2.24 ± 0.14^a^	1.86 ± 0.20^a^
RLgmcA	131.3 ± 11.3^a^	1.56 ± 0.05^b^	1.10 ± 0.18^b^
RLgmcA(pBBRgmcA)	135.5 ± 11.3^a^	2.12 ± 0.16^a^	1.80 ± 0.16^a^
WC	0	0	0.35 ± 0.09^c^

*All data are averages (±SEM) from ten independent plants*.

a, b, c *Different letters indicates the value is significantly different from that of the wild-type RL3841 control (one-way ANOVA, P < 0.05). WC, water control without inoculation*.

The authors apologize for these errors and state that this does not change the scientific conclusions of the article in any way. The original article has been updated.

## Publisher's Note

All claims expressed in this article are solely those of the authors and do not necessarily represent those of their affiliated organizations, or those of the publisher, the editors and the reviewers. Any product that may be evaluated in this article, or claim that may be made by its manufacturer, is not guaranteed or endorsed by the publisher.

