# Supplementary figures and images for: A GMC Oxidoreductase GmcA Is Required for Symbiotic Nitrogen Fixation in Rhizobium leguminosarum bv. viciae
Source: Front Microbiol. 2020 Mar 24;11:394. doi: 10.3389/fmicb.2020.00394 (PMC7105596; doi:10.3389/fmicb.2020.00394)

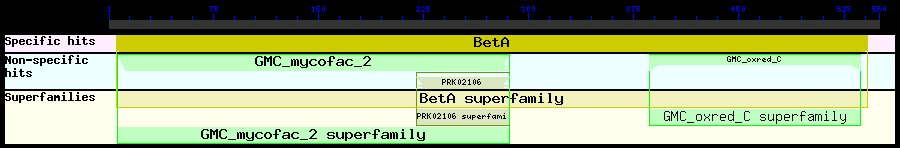

Supplement: FIGURE S1 — Analysis of the protein domains of GmcA in Rhizobium leguminosarum 3841. BetA, choline dehydrogenase or related flavoprotein; GMC_oxred_C, GMC oxidoreductase; GMC_mycofac_2, GMC family mycofactocin-associated oxidoreductase. [file Image_1.TIF]

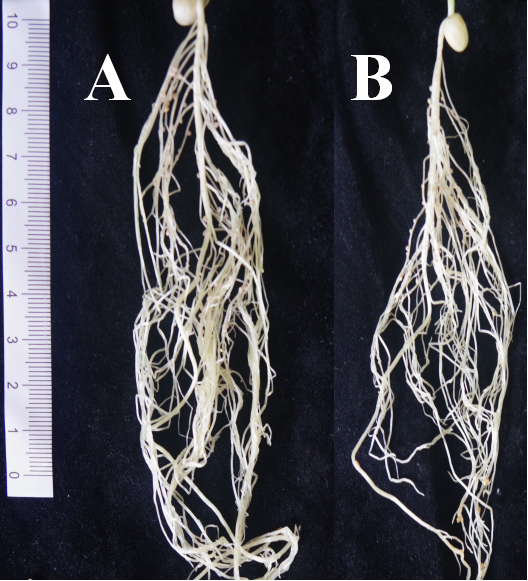

Supplement: FIGURE S2 — Plant growth test of the symbiotic ability of R. leguminosarum. (A) Control plant root inoculated with the wild type RL3841, (B) Plant root inoculated with RLgmcA. [file Image_2.TIF]
